# Emergency Separation of Extreme VLBW Omphalopagus Twins: Case Report

**DOI:** 10.1055/s-0042-1750134

**Published:** 2022-07-04

**Authors:** Waleed Burhamah, Amar Alnaqi, Yaqoub Jafar, Esmaeel Taqi

**Affiliations:** 1 Royal College of Surgeons in Ireland, Dublin, Ireland; 2 Department of Pediatric Surgery, Ibn Sina Hospital, Sabah Medical Center, Kuwait, Kuwait

**Keywords:** conjoined twins, bladder exstrophy, omphalopagus twins

## Abstract

The classification of conjoint twins is based according to the site of attachment. The challenges in management of such anomalies span the entire continuum of care from delivery to resuscitation to separation and finally discharge. Scheduled separation is ideal, occasionally the caring team is faced with no option but to perform an emergent separation. Omphalopagus is a type of conjoined twinning characterized by union of the peritoneal cavities through an infraumbilical abdominal wall defect. In this report we describe our experience with a successful emergency separation of extremely preterm omphalopagus twins.

This is the first case of conjoint twins in Kuwait, we highlight the challenges faced, stressing the importance of adhering to antenatal care as well as management by a multidisciplinary team.

## Introduction

Conjoined twins is a rare condition that usually arises in monozygotic twins and is commonly associated with conjoined organs and secondary anomalies. 1 The prevalence of conjoint twins is variable and has been estimated to be 1 in 50,000 to 1 in 100,000 births. In up to 70% of the cases the twins are females. 2 Conjoined twins are classified according to the site of attachment, with each case representing a unique entity. 3

In this report, we describe our experience with a successful emergency separation of extremely preterm omphalopagus twins. This is the first case of conjoint twins in Kuwait, we highlight the challenges faced and describe our approach in managing this case.

## Case Report

A 27-year-old (gravida 1) previously healthy mother gave birth to conjoined twins at 26 + 4 weeks via emergency cesarean section due to fetal distress. Antenatal ultrasound had shown monochorionic diamniotic twins with no evidence of associated anomalies. Upon recognition of the conjoint twinning the obstetric team undertook the following precautions: clamping the umbilical cord distally, as well as ensuring correct axis orientation of both babies to avoid twisting of the conjoint segment.

**Fig. 1:**
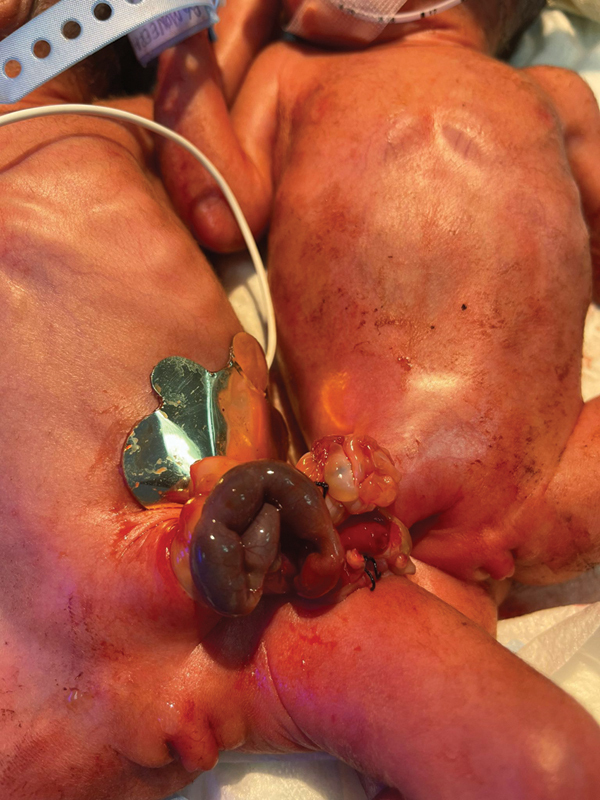
The conjoined twins upon initial assessment following birth. Note: the conjoined segment with a dusky segment of small bowel.

Upon birth the Apgar score was 4 and 7 at 1 and 5 minutes, respectively, for both babies. Both babies showed signs of respiratory distress and were intubated and placed on mechanical ventilation. Upon subsequent systemic assessment a cardiovascular examination was unremarkable, their vital signs were within normal range with no requirement of inotropes. Both babies had a ruptured omphalocele with entwined small bowel loops and a dusky segment of small bowel that was visible externally. They both had ambiguous genitalia—with well-formed labia majora, a deficient labia minora, and a single perineal opening. Attempt to cannulate the perineal opening with a Foley catheter failed in baby 1, but yielded urine in baby 2. Both babies had imperforate anus, exstrophy, and weighed 800 g each ( Fig. 1 ). An echocardiogram for both babies was performed and did not reveal any significant malformations. In both babies an ultrasound of the kidney, ureters, and bladder confirmed the bladder exstrophy with no additional anomalies.

The conjoined twins were taken to emergency surgery that lasted for 6 hours. Intraoperative examination of the conjoined parts showed segments of dusky small bowel and the umbilical cords, serving as points of attachment between the babies ( Fig. 1 ). Conjoined exstrophied bladders were identified in both babies. A decision was made to carry out a midline laparotomy as opposed to a transverse incision to accommodate the anticipated ostomies and facilitate the dissection of bladder plate

### Baby 1

A laparotomy revealed presence of the stomach, small bowel, and no evidence of large bowel.

**Fig. 2:**
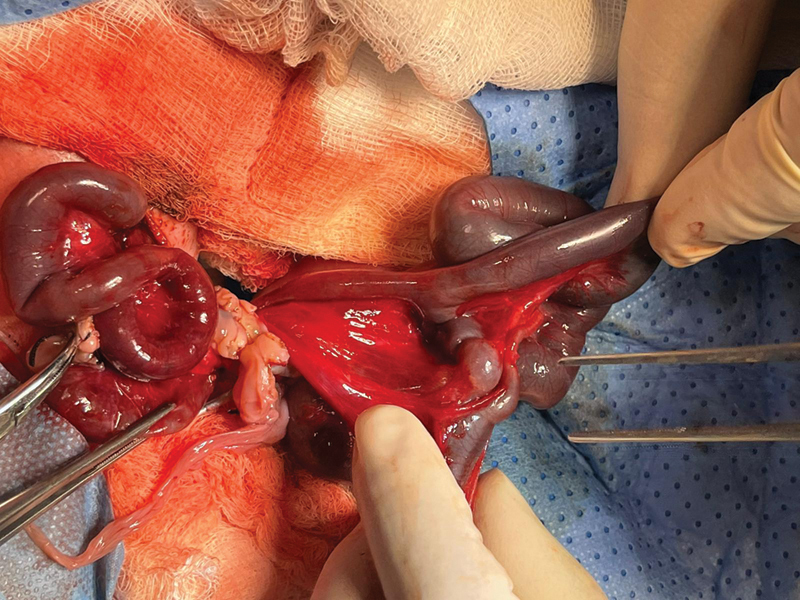
An intraoperative image demonstrating the small bowel of baby 1 ending distally in the anti-mesenteric boarder of the terminal ileum in Baby 2.

The small bowel ended distally in the antimesenteric border of the terminal ileum in baby 2 ( Fig. 2 ). An exstrophied bladder was identified with both ureters entering respectively in the exstrophied plates. A uterus and two ovaries were identified.

### Baby 2

The laparotomy showed presence of the stomach, small bowel, and large bowel. In addition, an exstrophied bladder with both ureters entering respectively in the exstrophied plates was identified. A rectovesical fistula joining the bladder neck was also seen. A uterus and two ovaries were identified.

**Fig. 3:**
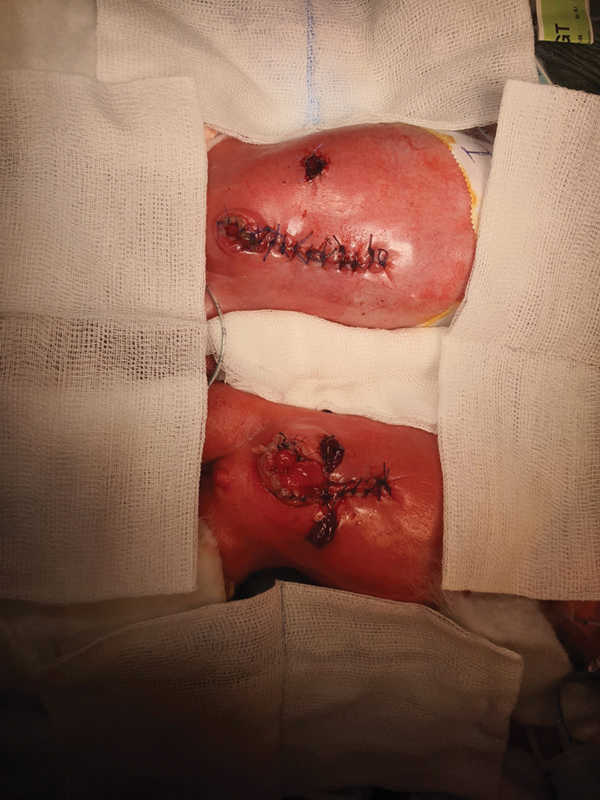
An immediate post-operative image of both babies. Note the end ileostomy in baby 1 and an end colostomy with a mucous fistula in baby 2. A vesicostomy was created in both babies.

During the surgery the twins underwent separation of the small bowel loops with resection of a dusky segment of ileum measuring 15 cm. An end ileostomy was matured in baby 1 and an end colostomy with a mucous fistula in baby 2. In both babies the conjoint bladders were separated and matured into a vesicostomy, and the omphalocele was closed ( Fig. 3 ). During separation of the conjoint bladders the main challenge was to safely identify and protect both ureters, this was achieved with meticulous dissection.

**Fig. 4:**
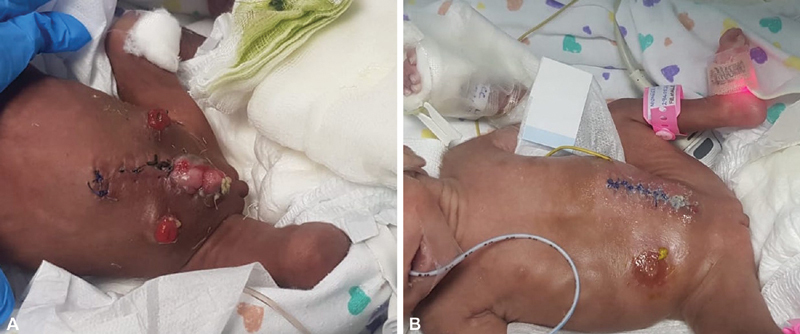
( **A** and **B** ) Illustrates a follow-up image at 5 months of age for both babies.

**Fig. 5:**
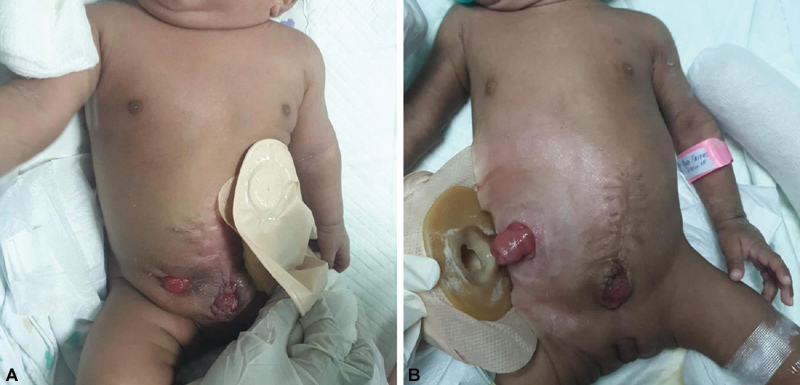
( **A** , **B** ) Illustrates a follow-up image of age for both babies.

Postoperatively, both babies were admitted to the intensive care unit for observation. An ultrasound abdomen showed no evidence of hydronephrosis, indicating no compromise to the ureter had occurred during dissection of the bladder plates. The recovery course was unremarkable. Recent follow-up at 5 months of age showed both babies successfully weaned off the mechanical ventilator, tolerating oral feeds with functioning ostomies ( Figs. 4A, B and 5A, B ) At 1 year of age both babies will undergo a delayed repair of the bladder exstrophy and an anterior pelvic osteotomy. Additionally, baby 2 will undergo an anorectoplasty.

## Discussion

In 1689, Konig described the first successful separation of conjoined twins. 4 Since then, a worldwide collaborative epidemiological study of the International Clearinghouse for Birth Defects Surveillance Programs, a total of 383 sets of conjoint twins were reported out of 26,138,837 births, giving a total prevalence of 1.47 per 100,000 births (95% confidence interval: 1.32–1.62). 3

Conjoined twins are classified according to site of attachment, with the most common being thoracopagus, craniopagus, and omphalopagus. 3 Omphalopagus is a type of conjoined twinning characterized by union of the peritoneal cavities through an infraumbilical abdominal wall defect. It represents 10 to 14% of all conjoined twins, 5 6 7 and can be associated with union of the intestinal tract, liver, and/or abnormalities of derivatives of the cloaca. There is a wide spectrum of presentations based on the degree of organ sharing, ranging from the minimal conjoined omphalopagus twinning to complex sharing of the liver and/or the biliary system. 8 9 A conjoined biliary tract is reported in up to 25% of omphalopagus twins. 10

Although extremely rare, the association of conjoined twinning with cloacal anomalies is recognized, and only a handful of cases describe an association with bladder exstrophy. 11

The type of placentation that is commonly associated with conjoined twins is monochorionic monoamniotic. Monozygotic twins originate from the early division of a single embryo. They can be dichorionic diamniotic (30–40%), monochorionic diamniotic (60–70%), or monochorionic monoamniotic. In our case, the conjoined twins were diamniotic, which represent the minority of published cases in the literature. 12 13 14 15

The management of conjoined twins poses a challenge not only to the pediatric surgeon but the entire team of physicians. The challenges span the entire continuum of care from delivery to resuscitation to separation and finally discharge. The decision on timing of separation is critical in achieving success. Scheduled separation is ideal, it allows for a detailed radiological evaluation of the shared organs, presence of anomalies, presence and extent of cross-circulation, and subsequently allows planning of the surgical approach. 16 This emphasizes the importance of adhering to antenatal care as well as management by a multidisciplinary team.

Occasionally, the caring team is faced with no option but to perform an emergent separation. This may be indicated in cases of stillbirth of one of the babies, 17 rupture of the omphalocele, 18 gastroschisis, 19 trauma to the joint structures, 12 and/or intestinal obstruction. 20 21

## Conclusion

Conjoined twins is a rare condition, presenting with an armamentarium of associated anomalies. In the presence of a life-threatening event, for example, bowel ischemia, emergency separation is indicated. Management in a tertiary center by a multidisciplinary team should be the standard of care.

## Notes

New Insights and the Importance for the Pediatric Surgeon The management of conjoined twins poses a challenge to the entire caring team of physicians. The challenges span the entire continuum of care from delivery to resuscitation to separation and finally discharge. Management by a multidisciplinary team is essential.
