# Erratum to: Emergency Separation of Extreme VLBW Omphalopagus Twins: Case Report

**DOI:** 10.1055/s-0042-1755532

**Published:** 2022-08-23

**Authors:** Waleed Burhamah, Amar Alnaqi, Yaqoub Jafar, Esmaeel Taqi

**Affiliations:** 1 Royal College of Surgeons in Ireland, Dublin, Ireland; 2 Department of Pediatric Surgery, Ibn Sina Hospital, Sabah Medical Center, Kuwait, Kuwait

The Publisher wishes to inform that the page range of the above-mentioned article has been updated. DOI of the article is 10.1055/s-0042-1750134 . The corrected page range is e111-e114.

